# Post-enucleation socket syndrome—a novel pathophysiological definition

**DOI:** 10.1007/s00417-022-05648-z

**Published:** 2022-04-02

**Authors:** Alexander C. Rokohl, Adam Kopecky, Marc Trester, Philomena A. Wawer Matos, Keith R. Pine, Ludwig M. Heindl

**Affiliations:** 1 Department of Ophthalmology, Faculty of Medicine and University Hospital of Cologne, University of Cologne, Kerpener Straße 62, 50924 Cologne, Germany; 2 Center for Integrated Oncology (CIO), Aachen-Bonn-Cologne-Dusseldorf, Cologne, Germany; 3 Ophthalmology Clinic, University Hospital Ostrava, Ostrava, Czech Republic; 4 Department of Craniofacial Surgery, Faculty of Medicine, University of Ostrava, Ostrava, Czech Republic; 5 Trester-Institute for Ocular Prosthetics and Artificial Eyes, Cologne, Germany; 6 School of Optometry and Vision Science, University of Auckland, Auckland, New Zealand

**Keywords:** Post-enucleation socket syndrome, Anophthalmia, Ocular prostheses, Anophthalmic socket, Prosthetic eye, Enucleation

## Abstract

**Background:**

The last definition of the post-enucleation socket syndrome (PESS) by Tyers and Collin—formulated almost 40 years ago in 1982—is predominantly based on the clinical characteristics and does not include the insights of newer studies into the pathophysiological mechanism of the PESS.

**Methods:**

A systematic PubMed literature review regarding the pathophysiological mechanism of the PESS was performed, and results were comprised to give an overview of the current knowledge of the PESS including the exact pathophysiological mechanism.

**Results:**

The primarily postulated pathophysiological mechanism of the PESS was the atrophy of orbital tissues, especially of fat, resulting in variable clinical findings. Newer studies using high-resolution computed tomography and magnetic resonance imaging or performing histopathological analyses found no orbital fat atrophy but rather a rotatory displacement of the orbital tissues from superior to posterior and from posterior to inferior together with the retraction of the extraocular muscles and a possible volume loss of the orbital implant by resorption if it is manufactured from hydroxyapatite. PESS results in a backward tilt of the superior fornix, a deep superior sulcus, a pseudo-ptosis, a lower eyelid elongation and laxity, a shallower inferior fornix, as well as enophthalmos and may lead to an inability of wearing ocular prostheses.

**Conclusions:**

A novel and comprehensive definition of the PESS is proposed: PESS is a multifactorial and variable syndrome caused by a rotatory displacement of orbital contents together with the retraction of the extraocular muscles and possible resorption of the orbital implant if it is manufactured from hydroxyapatite.

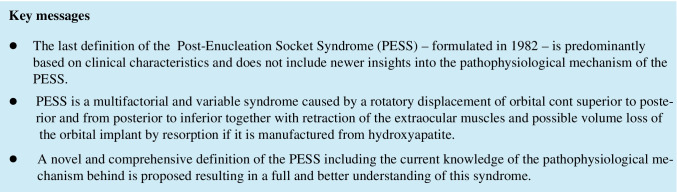



## Introduction

The enucleation of an eye is a life-changing event for every patient with a high psychological impact and emotional burden [1–3]. In addition to the functional disability with vision and visual field loss, cosmetic and aesthetic aspects are one of the most important concerns after enucleation [1–3]. The PESS is one of the main reasons for a bad appearance with a prosthetic eye [1–5].

**Fig. 1:**
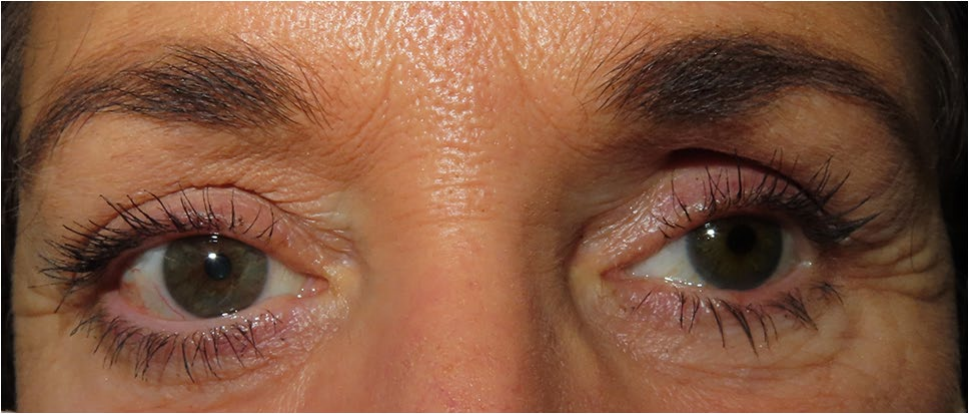
A 51-year-old female patient with PESS on the left side. Clinical findings include significant volume displacement with a deep upper eyelid sulcus, enophthalmos of the artificial eye, backward tilt, and upward and left gaze of the prosthesis

In 1982, the term PESS was introduced by Tyers and Collin for the first time [6, 7]. PESS was originally characterized as a constellation of variable clinical findings including a deep upper eyelid sulcus, ptosis, enophthalmos of the artificial eye, and lower eyelid elongation and laxity (Fig. 1) [6, 7]. Tyers and Collins observed these clinical changes mostly beginning already in the first 2 years after enucleation [6, 7]. The clinical findings were more pronounced over time and also if the orbital implant was too small at the time of surgery or no implant was used [6, 7].

The primarily postulated pathophysiological mechanism of the PESS only based on these clinical findings were anatomical changes of the anophthalmic socket over time, namely atrophy of orbital tissues and more precisely of the orbital fat [6]. The last definition of the PESS by Tyers and Collin—formulated almost 40 years ago in 1982—is predominantly based on the clinical characteristics and does not include the current knowledge about the pathomechanism of the PESS [6, 7]. Although there were some studies providing insights into the pathophysiological mechanism of the PESS since 1982 [8–10] (Table 1), an updated definition of the PESS that includes the current knowledge is still lacking until today. For a full and better understanding and in order to formulate an updated definition of the PESS, a deeper and more comprehensive look into the pathomechanism of the post-enucleation socket syndrome is necessary, especially in conjunction with current knowledge regarding this syndrome affecting the quality of life [1–3, 11].

**Table 1:** Overview of studies investigating the pathophysiological mechanism of the PESS

Study	Publication date	Investigation method	Novel findings
Tyers et al. [6]	1982	Clinical observation	Variable clinical findings including a deep upper eyelid sulcus, ptosis, enophthalmos of the artificial eye, and lower eyelid elongation and laxity
Smit et al. [8]	1990	Computed tomography (CT)	Rotatory displacement of the orbital tissues from superior to posterior and from posterior to inferior, enophthalmos, a sagged and retracted superior muscle complex correlating with a deepening of the superior sulcus, a forward displacement of the posterior positions of Tenon’s capsule, a downward and forward redistribution of the orbital fat, and an upward displacement with retraction of the inferior rectus muscle
Detorakis et al. [9]	2003	Magnetic resonance imaging (MRI)	Muscle contraction and retraction resulting in a significantly reduced muscle length
Han et al. [10]	2021	Histopathological analyses	Shrinking of mammalian bone-derived hydroxyapatite orbital implants by osteoclastic activity

## Pathophysiological mechanism of the PESS

**Fig. 2:**
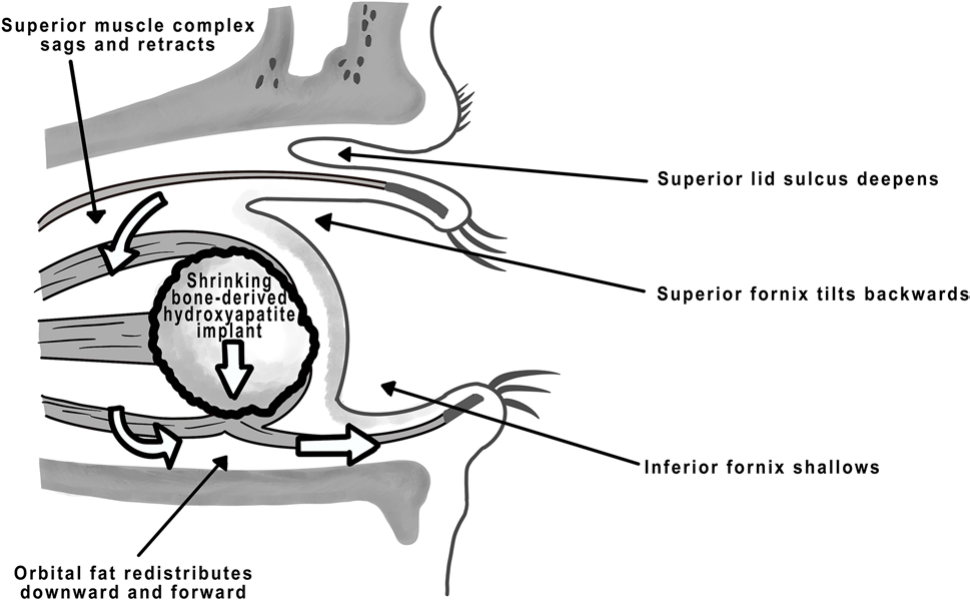
Orbital tissue alterations and shrinking of mammalian bone-derived hydroxyapatite orbital implants by osteoclastic activity in the course of the PESS [7]

Orbital volume loss by atrophy of orbital tissues was stated as the cause of the PESS for a long time [6]. However, in 1990, Smit et al. [8] performing high-resolution computed tomography (CT) examinations of anophthalmic sockets, found no atrophy of any orbital tissues but a redistribution of orbital contents. In fact, the pathophysiological mechanism behind the PESS seems to be a rotatory displacement of the orbital tissues from superior to posterior and from posterior to inferior (Fig. 2) [8]. CT showed enophthalmos, a sagged and retracted superior muscle complex correlating with a deepening of the superior sulcus, a forward displacement of the posterior positions of Tenon’s capsule, a downward and forward redistribution of the orbital fat, and an upward displacement with retraction of the inferior rectus muscle (Fig. 2) [8]. In 2003, Detorakis et al. analyzed the functional anatomy of anophthalmic sockets using magnetic resonance imaging (MRI) and confirmed the previous CT results. This study showed also no alterations in the volumes of the orbital fat or the extraocular rectus and oblique muscles but revealed muscle contraction and retraction resulting in a significantly reduced muscle length [9].

While previous studies did not find any evidence for orbital volume loss in the development of the PESS, in 2021, Han et al. detected the first time a reason for orbital volume loss contributing to the PESS potentially [10]. A shrinking of mammalian bone-derived hydroxyapatite orbital implants by osteoclastic activity was observed [10]. While the patients in the study of Smit et al. had orbital implants made of acrylic, Detorakis et al. included also patients with mammalian bone-derived hydroxyapatite implants (Molteno M-sphere) [9], but they did not analyze potential volume changes of the orbital implants [9]. A reason for this might be, besides the study design, the fact that mammalian bone-derived hydroxyapatite orbital implants were not in widespread use at the time. The first was introduced by Arthur Perry in 1985, just 5 years before Smit’s study. To summarize, the shrinking of mammalian bone-derived hydroxyapatite orbital implants by osteoclastic activity is a novel finding. Although mammalian bone-derived hydroxyapatite orbital implants are used rather rarely, they can have a significant role in orbital volume loss in patients presenting with the PESS, at least if this type of implant is used. Since resorption of synthetic porous hydroxyapatite implants—even though outside the orbit—is also described in the literature [12], these findings suggest the use of orbital implants made from polyethylene, acrylic, polymethylmethacrylate (PMMA), or silicone. The use of these materials might reduce shrinking or resorption of the orbital implant and prevents therefore also volume loss potentially contributing to the PESS.

**Fig. 3:**
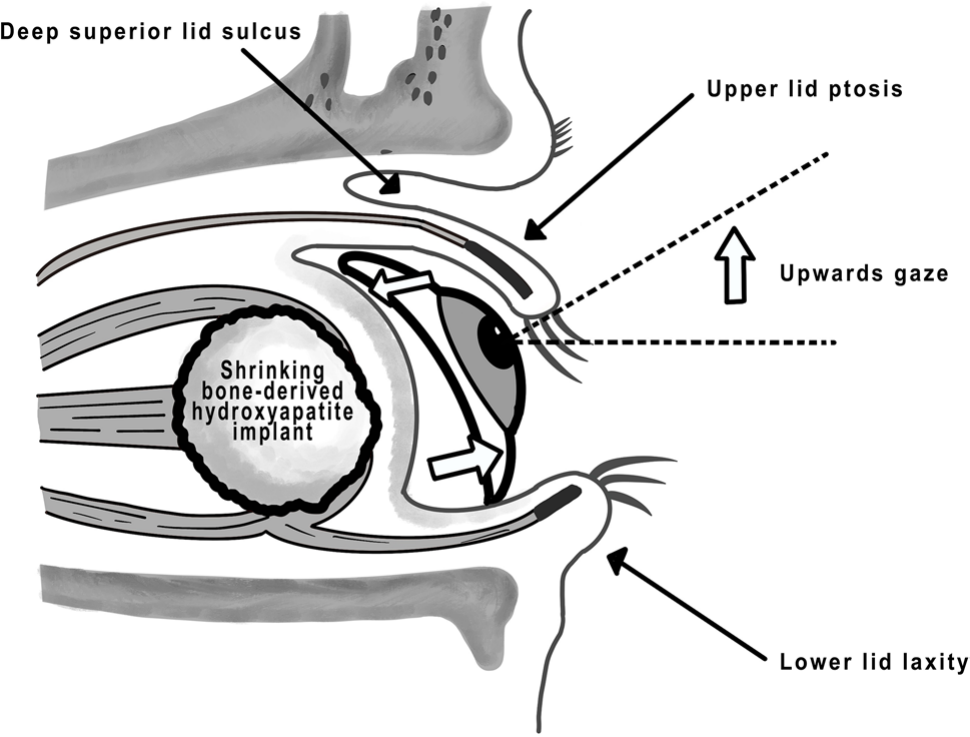
Clinical elements of the PESS and the impact on the ocular prosthesis. The prosthesis tilts backward, gazes upward, and puts forward pressure on the lower eyelid [7]

All these alterations of anophthalmic sockets induce a shallowing of the inferior fornix as well as a backward tilt and upwards gaze of the prosthesis with forward pressure on the lower eyelid (Fig. 3) [7]. This in turn causes reduced support of the upper eyelid leading to a deepening of the superior sulcus and a reduced superior eyelid crease resulting in pseudo-ptosis [7]. Furthermore, the forward pressure on the lower eyelid causes it to stretch resulting in an increased lower eyelid elongation and laxity [7]. Even worse, this vicious cycle of the PESS may lead to reduced upper eyelid motility including lagophthalmos, upper and lower lid entropion or ectropion, prosthesis instability, and eventually even to an inability of wearing ocular prostheses [7].

## Post-evisceration socket syndrome or anophthalmic socket syndrome?

**Fig. 4:**
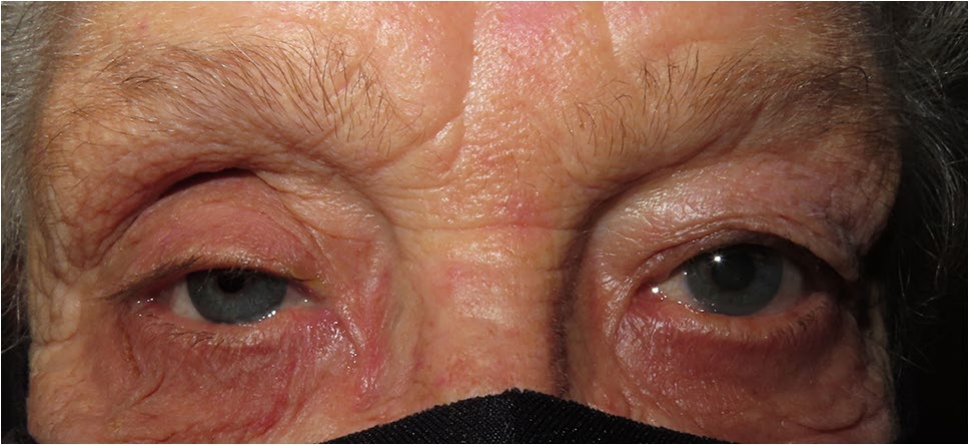
A 72-year-old female patient with post-evisceration socket syndrome on the right side. Clinical findings include similar to the PESS significant volume displacement with a deep upper eyelid sulcus, enophthalmos of the artificial eye, ptosis, as well as lower eyelid elongation and sinking

Since clinical signs of the PESS are often also observed after evisceration of an eye, some studies use the terms post-enucleation or evisceration socket syndrome, post-evisceration socket syndrome, or more general anophthalmic socket syndrome [13–18]. However, only the pathomechanism of the PESS is already investigated in detail and confirmed with high-resolution imaging of anophthalmic sockets. In the clinical experience of the authors, there seems to exist a post-evisceration socket syndrome having the same clinical signs as the PESS (Fig. 4). These clinical signs seem to be variable and may include a deep upper eyelid sulcus, ptosis, enophthalmos of the artificial eye, and lower eyelid elongation and laxity (Fig. 4). However, these clinical signs and the pathophysiological mechanism behind the post-evisceration socket syndrome have to be investigated in detail in larger future studies, and only then can be decided whether the PESS and the post-evisceration socket syndrome have the same pathomechanism or not and whether both syndromes should be summarized under the term anophthalmic socket syndrome. Until then, the PESS should be seen and defined as an independent syndrome.

## Post-enucleation socket syndrome—a novel definition

In summary, based on the results of the previous studies investigating the pathophysiological mechanism of the PESS, we propose—for the first time since 1982—a comprehensive definition of the PESS:

The PESS is a multifactorial and variable syndrome caused by a rotatory displacement of orbital contents from superior to posterior and from posterior to inferior together with retraction of the extraocular muscles and possible volume loss of the orbital implant by resorption if it is manufactured from hydroxyapatite. These orbital changes occur most rapidly in the months immediately following eye loss but continue at a slower pace for the rest of the patient’s life and were more pronounced if the orbital implant was too small at the time of surgery or no implant was used. PESS results in a backward tilt of the superior fornix, a deep superior sulcus, a pseudo-ptosis, a lower eyelid elongation and laxity, a shallower inferior fornix, as well as enophthalmos and may lead to an inability of wearing ocular prostheses.
